# A Treatise on the Physiological and Moral Management of Infancy

**Published:** 1840-07

**Authors:** 


Art. I.—*A Treatise on the Physiological and Moral Management of Infancy.* By Andrew Combe, M.D., Physician Extraordinary to the Queen, &c.—*Edinburgh,* 1840. 12mo, pp. 375.

After a careful perusal of this little volume from beginning to end, we do not hesitate to pronounce it to be one of the most valuable and most important works that has issued from the medical press for years. We make this statement in reference as well to the matter of the book as to the manner in which it is written, and also the probable extent to which it will be read. There may be much information in a work which is nevertheless of little value from the unattractive way in which it is conveyed; and it does not always happen that books which are both instructive and well written become favorites with the public, and are in consequence perused by a vast number of persons. The works on popular medicine, however, hitherto written by Dr. Combe, have possessed all the qualities calculated to ensure that highest and best popularity which consists in the perusal and study of them by a large proportion of the well-instructed and well-disposed in every rank of society; and when we see that the present volume possesses all the excellences of its precursors, and the advantage of their reputation also, we have no doubt of the extensive popularity that awaits it. We learn by an advertisement of Dr. Combe's publisher, inserted at the end of this treatise, that no less than 13,000 copies of his "Principles of Physiology applied to Health" have been sold in Great Britain alone (and double that number in America) since its publication in 1834; and that upwards of 5000 copies of the "Physiology of Digestion," published two years subsequently, have been similarly disposed of. A popularity like this, which we believe to be unequalled among medical works, while it is the highest reward of an author's labours, imposes upon him a proportional amount of responsibility as to the character of the future works he may lay before the public, since his opinions, right or wrong, are certain of influencing, for good or for evil, a large portion of the community. And the responsibility is still further enhanced when the subject discussed is, as in the treatise before us, one of the very first importance to the physical and moral condition,—to the life, health, and happiness of mankind. The terms in which we have, in our opening sentence, briefly characterized this new production of Dr. Combe sufficiently evince our opinion as to the manner in which the author has, in its pages, acquitted himself of his high obligations, and preferred fresh claims to the consideration and gratitude of his contemporaries.

And yet it is not so much from the originality of the facts and doctrines communicated as from their completeness and the peculiar manner in which they are communicated, that this small treatise so greatly excels all that have preceded it in the same line. This is admitted by the author himself, who tells us, in his preface, that "it is in the habitual application of *principle* to the inculcation and advancement of knowledge, more than in any absolute novelty of detail," that he expects it to be found deserving of notice. It is certainly most true, as is also stated by Dr. Combe, that most of the works of the kind hitherto published, however excellent in other respects, "lose much of their value and usefulness from presenting their rules and admonitions as so many abstract individual opinions, and omitting to connect them with the physiological laws or principles on which they are based, and according to which their effects are produced." Accordingly it has been, he says, his constant endeavour, in the present as in his former works, "to allow as little as possible to rest on mere human opinion, but to show a foundation for every rule, precept, and injunction, in the laws of the human constitution, and consequently in the will of the Creator." How completely he has accomplished his intentions, with what a rigid adherence to his principle, with what sustained interest of exposition, and with what fertility of results, will be admitted by every reader. We regret extremely that the late period of the quarter at which this volume reached us puts it out of our power to do more than to pass this general judgment on its merits, and to give a mere catalogue of its contents. We hope, however, to be able to return to it on some future occasion, and to enrich our pages with some of its more important details, which, although expressly calculated for the general reader, will be found no less interesting and valuable to the profession. Indeed we are persuaded, from long experience and observation, that there are few members of the profession who will not derive some benefit from this little work; while to young practitioners, and to all enlightened parents, it cannot fail to prove of inestimable value. The last chapter, "On the Moral Management of Infancy," humble as are its pretensions, we venture to recommend to the notice of the instructors of youth of every degree, to our moral teachers however elevated, and even to our metaphysicians however learned, as fraught with truths of the most momentous kind, which will probably be new to many of them, and which cannot fail, if candidly considered and honestly acted on, to lead to practical results of the highest import to human happiness. In it the author touches lightly, but with a masterly hand, on that chord, mostly overlooked by our philosophers, which unites so harmoniously the intellectual and moral with the physical nature of man, and the due recognition and just appreciation of which are indispensable to our progress in real metaphysics, and to the establishment of all rational instruction.

The work is divided into fifteen chapters, with the following summary headings, which however but imperfectly convey the extent and variety of the matter contained in them respectively:—1. Introductory Explanation. 2. Extent of Mortality in Infancy. 3. Sources of Disease in Infancy. 4. Delicacy of Constitution in Infancy. 5. Conditions in the Mother affecting the health of the Child. 6. Constitution of the Infant at birth. 7. The Nursery and conditions required in it. 8. Management of the Infant immediately after birth. 9. Food for the Infant at birth. 10. The choice, properties, and regimen of a Nurse. 11. Artificial Nursing and Weaning. 12. Cleanliness, Exercise, and Sleep in early Infancy. 13. Management during Teething. 14. Management from the time of Weaning to the end of the second year. 15. The Moral Management of Infancy.

The work is printed in a neat but unexpensive style. It is dedicated by the author to his friend Sir James Clark, in terms of warm affection and not unbecoming praise. All who have had opportunities of judging of Sir James as a professional brother and practitioner, and every reader of his classical works on Climate and Consumption, will readily assent to the justness of Dr. Combe's estimate of his merits, and of his especial claims to consideration as a successful labourer in the highest and most philosophical department of medicine—the hygiènic treatment of man.

"For many years (says Dr. Combe) not only have you taken a deep and active interest in the improvement of medical education, and in elevating the character, extending the scope, and increasing the usefulness of the profession; but acting on the same principles which I have endeavoured to enforce, you have rendered no small service to science by your instructive exposition of the manner in which fatal disease of the lungs so often and so insidiously originates in apparently trifling causes connected with disregard of the ordinary laws of health. You have further shown that when medicine shall be cultivated in a more liberal and comprehensive spirit, and its principles be recognized as furnishing the only solid foundation for a proper system of physical, moral, and intellectual education, it will become one of its noblest uses, and, I may add, one of its greatest privileges to be instrumental not more in the prevention of disease and suffering than in largely contributing to the general happiness and permanent advancement of the human race."

